# The Ketogenic Diet Is an Effective Adjuvant to Radiation Therapy for the Treatment of Malignant Glioma

**DOI:** 10.1371/journal.pone.0036197

**Published:** 2012-05-01

**Authors:** Mohammed G. Abdelwahab, Kathryn E. Fenton, Mark C. Preul, Jong M. Rho, Andrew Lynch, Phillip Stafford, Adrienne C. Scheck

**Affiliations:** 1 Neuro-Oncology Research, Barrow Neurological Institute® of St. Joseph's Hospital and Medical Center, Phoenix, Arizona, United States of America; 2 Neurosurgery Research, Barrow Neurological Institute® of St. Joseph's Hospital and Medical Center, Phoenix, Arizona, United States of America; 3 Pediatric Epilepsy Research, Barrow Neurological Institute® of St. Joseph's Hospital and Medical Center, Phoenix, Arizona, United States of America; 4 Nutricia Advanced Medical Nutrition, Danone Research, Centre for Specialised Nutrition, Liverpool, United Kingdom; 5 AZ Biodesign, Center for Innovations in Medicine, Arizona State University School of Life Sciences, Tempe, Arizona, United States of America; Columbia University, United States of America

## Abstract

**Introduction:**

The ketogenic diet (KD) is a high-fat, low-carbohydrate diet that alters metabolism by increasing the level of ketone bodies in the blood. KetoCal® (KC) is a nutritionally complete, commercially available 4∶1 (fat∶ carbohydrate+protein) ketogenic formula that is an effective non-pharmacologic treatment for the management of refractory pediatric epilepsy. Diet-induced ketosis causes changes to brain homeostasis that have potential for the treatment of other neurological diseases such as malignant gliomas.

**Methods:**

We used an intracranial bioluminescent mouse model of malignant glioma. Following implantation animals were maintained on standard diet (SD) or KC. The mice received 2×4 Gy of whole brain radiation and tumor growth was followed by *in vivo* imaging.

**Results:**

Animals fed KC had elevated levels of β-hydroxybutyrate (p = 0.0173) and an increased median survival of approximately 5 days relative to animals maintained on SD. KC plus radiation treatment were more than additive, and in 9 of 11 irradiated animals maintained on KC the bioluminescent signal from the tumor cells diminished below the level of detection (p<0.0001). Animals were switched to SD 101 days after implantation and no signs of tumor recurrence were seen for over 200 days.

**Conclusions:**

KC significantly enhances the anti-tumor effect of radiation. This suggests that cellular metabolic alterations induced through KC may be useful as an adjuvant to the current standard of care for the treatment of human malignant gliomas.

## Introduction

Malignant brain tumors are a devastating disease with a high mortality rate. These tumors do not have defined boundaries and complete surgical removal is virtually impossible. In addition, the intrinsic heterogeneity and genetic instability in these tumors results in cells resistant to therapy. Thus, even after surgery, radiation and chemotherapy these tumors typically recur, leading to patient mortality and an average survival of approximately 1.5 years [1]. Increased survival of brain tumor patients requires the design of new therapeutic modalities, especially those that enhance currently available therapies and/or limit tumor growth. Advances in our understanding of the biology of these tumors has led to an increase in the number of targeted therapies in preclinical and clinical [2]–[4]. While these therapies may prove somewhat effective, the heterogeneity of this tumor often precludes the targeted molecules from being found on all cells in the tumor thus reducing the efficacy of these treatments. In contrast, one trait shared by virtually all tumor cells is altered metabolism.

Metabolic dysregulation of cancer cells was first described in the 1950s by Otto Warburg, who identified differences in glucose uptake and production of lactate between non-neoplastic and neoplastic cells, now referred to as the “Warburg Effect” [5]. The “Warburg Effect” refers to the tumor's use of aerobic glycolysis to provide energy as well as biomolecules for growth regardless of the availability of oxygen. Dysregulation of genes involved in glycolysis and glycolytic transport to the mitochondria of tumor cells has been reported, as have alterations to the mitochondria themselves [6]. Metabolic dysregulation can also be a result of loss of p53 and subsequent upregulation of serine-threonine protein kinase (Akt) which can lead to the production of an overabundance of ATP [7]. Because all cancers share metabolic dysregulation and unregulated production of energy due to these or other mechanisms, a therapy that exploits this trait is likely to have a broader impact than an individual targeted therapy. The ketogenic diet alters cellular metabolism and thus may have a broad impact on overall tumor growth [8]–[12].

We and others have demonstrated that the use of a ketogenic diet and/or caloric restriction causes a reduction in blood glucose, an elevation in blood ketones and extends life in mouse models of malignant brain tumors. In our previous work [13] we demonstrated that an approximate 6∶1 (fat∶ protein+carbohydrates) rodent ketogenic diet (KD; Bioserv F3666 diet, Bioserv, Frenchtown, NJ) not only increases survival, it also reduces reactive oxygen species (ROS) and alters the expression of genes involved in oxidative stress. This work also showed that overall gene expression in the tumor from animals fed a ketogenic diet was shifted more towards the gene expression seen in non-tumor-containing tissue from animals fed either the ketogenic diet or standard diet. Seyfried and co-workers have used a ketogenic diet and/or caloric restriction to demonstrate that elevating ketones prolongs survival in additional mouse models of glioma [14]–[16]. Since radiation following surgery has been shown to be the most effective adjuvant therapy for patients with high-grade gliomas, we analyzed the effect of a ketogenic diet on the anti-tumor activity of radiation. For these studies we used Ketocal® (SHS International Ltd, Liverpool L7 9PT, UK), a nutritionally complete powdered product used to administer the classical (4∶1) ketogenic diet for children over 1 year of age. We found that Ketocal® (KC) fed *ad libitum* significantly increased survival in both untreated animals and in animals treated with radiation above that seen with animals fed a standard diet alone or in combination with radiation.

There have been many investigations on the use of formulations of the ketogenic diet for the treatment of various neurodegenerative diseases due, at least in part, to its neuroprotective properties [17]. In addition, alternative metabolic treatments including long and short-term fasting and/or calorie restriction have been studied. [18]. Although others have investigated the use of varying formulations of the ketogenic diet in glioma [19], to the best of our knowledge, this study is the first to investigate the efficacy of this formulation of the ketogenic diet in combination with radiotherapy for the treatment of malignant glioma.

## Results

### KetoCal® Alone Prolonged Survival Following Tumor Implantation

**Figure 1:**
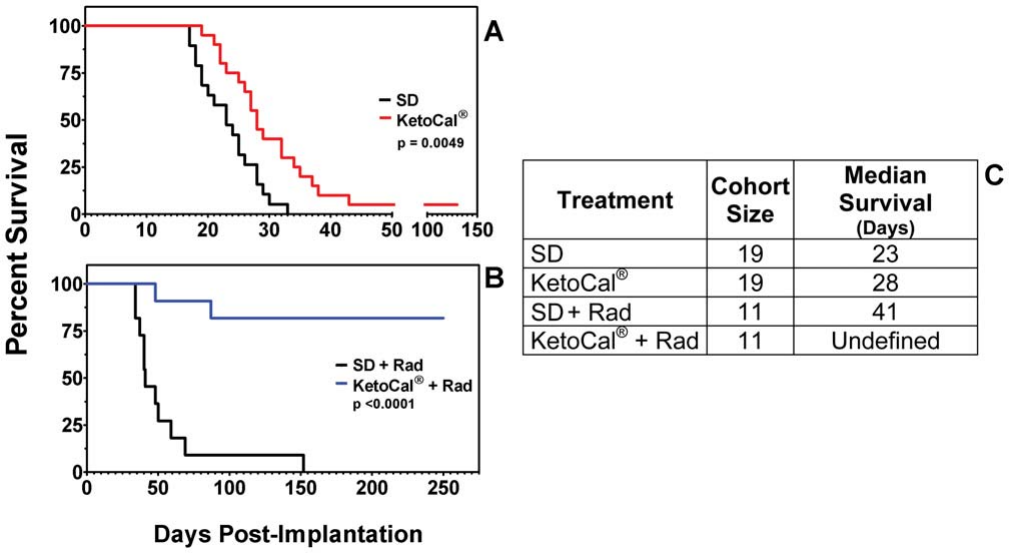
KetoCal® enhances survival of glioma-bearing mice. Kaplan-Meier plot of survival in KC versus SD (A), radiation versus KC plus radiation (B). Animals on KC survived significantly longer when treated with KC alone (p<0.005), or when combined with radiation (p<0.0001). Results are a combination of (A) 4 separate experiments and (B) 2 separate experiments.

Kaplan-Meier analyses of the survival data (Figure 1A) demonstrated a statistically significant (p<0.005) difference in median survival between animals fed SD (23 days) versus those fed KC (28 days). This increased survival was comparable to our previously reported results obtained on a smaller cohort of animals fed a ∼6∶1 (fat∶carbohydrate+protein) rodent ketogenic diet (Bio-Serv F3666 diet) [13]; however, in that experiment the maximum survival of animals fed the rodent ketogenic diet was only 5 days longer than the maximum survival of animals on the standard diet. In this report, maximum survival of animals fed SD was 33 days compared to 43 days in the group maintained on KC. One animal on KC alone was apparently cured of its tumor as evidenced by the loss of bioluminescent signal. On day 101 the animal was put back on standard rodent chow with no evidence of tumor re-growth for an additional 200 days, at which time the animal was sacrificed and had no histological evidence of disease.

### KetoCal® plus Radiation Treated Animals Were Apparently Cured of Implanted Tumor

**Figure 2:**
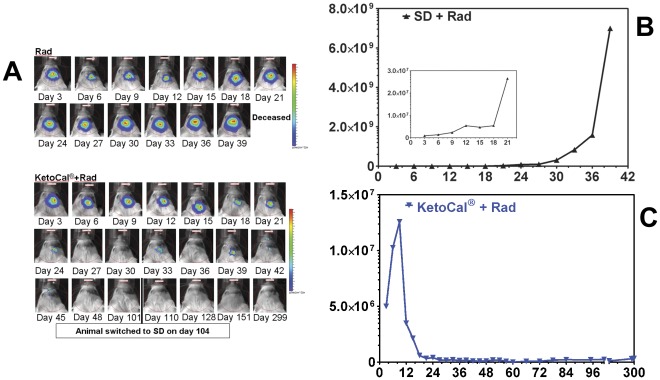
KetoCal® plus radiation treated animals experienced a loss of bioluminescence. (A) Radiation and KetoCal® plus radiation animal imaged every three days. Color scheme represents signal intensity with red representing the highest intensity photon count. (B) (C) Bioluminescent signal plotted as *in vivo* photon count versus days post-implantation.

It is unlikely that any new treatment will be tried in patients without some form of additional standard therapy. We therefore tested KC in addition to radiation to determine if the effect of the two treatments would be more than additive. Nine out of the 11 animals treated with KC in combination with radiation were apparently cured of their implanted tumor (Figure 1B). The *in vivo* imaging data from one representative animal treated with radiation alone and one treated with radiation and KC is shown in Figure 2. After an initial period of slower growth (Figure 2B, inset), there was rapid tumor growth in the animals fed SD and treated with radiation until the animal succumbed to the tumor (Figure 2A, B) with a final photon count of 6.995×10^9^ p/sec/cm^2^/sr on day 39 following tumor implantation. In the animal treated with radiation and KC the presence of growing tumor can be seen for the first 1.5–2 weeks following implantation, reaching a maximum bioluminescent signal of 1.258×10^7^ p/sec/cm^2^/sr on day 9 following tumor implantation. This was followed by a near exponential decline that approaches background levels 60 days following implantation. Bioluminescence remained undetectable and on day 104 post-implantation the 9 surviving animals treated with radiation and KC were switched from KC to the standard rodent chow. There was no detectable recurrence of tumor as demonstrated by the continued absence of detectable bioluminescent signal. The animals were sacrificed on day 299. Histological evidence upon necropsy using hematoxylin and eosin (H&E) staining of brain tissue from the apparently cured KC plus radiation animals showed no evidence of tumor cells in or near the area of implantation (data not shown).

Survival data from SD alone, KC alone, SD+radiation, and KC+radiation were examined for interaction effects using Cox Proportional Hazards. The p-value for radiation and KC is p = 1.03×10^−11^ vs. SD. The implication being that there is a profound enhancing (by mean survival) effect of radiation with KC vs. SD alone. There is also an enhancing effect of radiation with SD (p = 8.38×10^−2^) but the effect is many orders of magnitude less. Thus, we propose that a more than additive and highly positive survival effect is seen through the KC diet and adjuvant radiation therapy.

### Animals Fed KetoCal® Exhibit Elevated βHB levels

**Figure 3:**
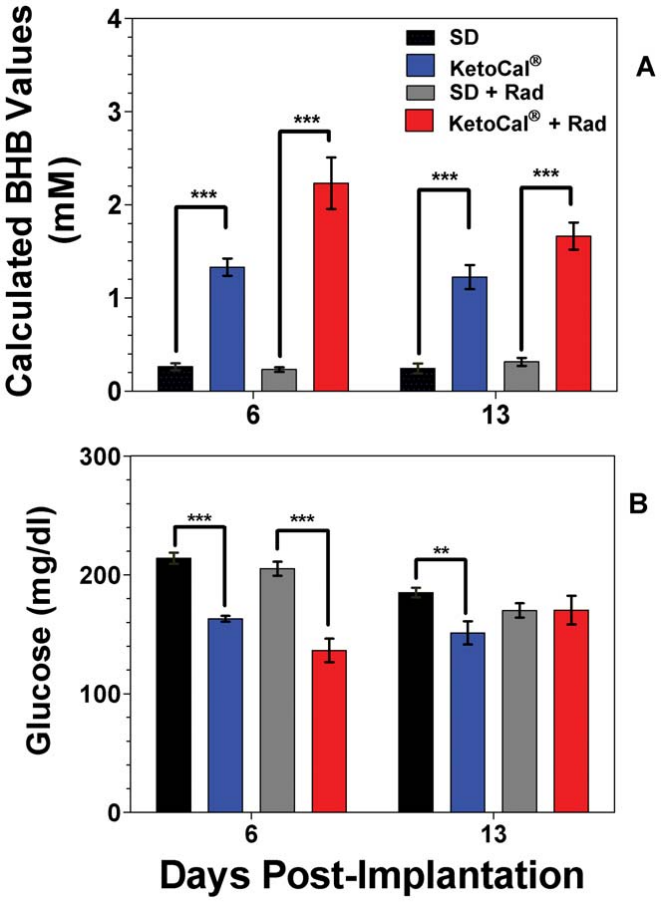
Blood ketone and glucose levels. Measurements of animal ketone and glucose levels show higher βHB blood levels in animals treated with KetoCal®. (A) βHB levels (B) Blood glucose measurements. ** = p<0.001; *** = p<0.0001.

Animals fed KC had a statistically significant increase in blood βHB levels (Figure 3A) both 6 and 13 days post-implantation. The greatest increase in βHB levels was seen in animals given adjuvant radiation therapy. However, increased βHB levels did not correlate with a decrease in blood glucose levels. Glucose levels were significantly lower in the KC and KC plus radiation groups on day 6 (p<0.0001) than SD and SD plus radiation; and only in the KC group on day 13 (p<0.001) when compared to SD.

**Figure 4:**
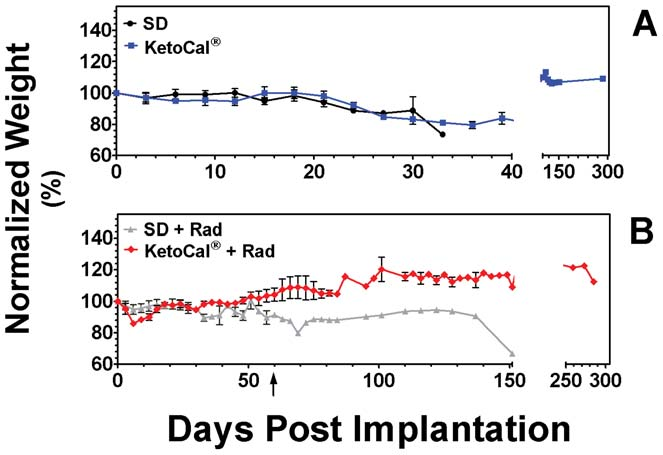
Animal weights. Weight measurements were taken every 3 days. Graph shows animals weights normalized to the average starting weight of each group on day zero. (B) Arrow denotes a long-term survivor 60 days following tumor implantation in the SD plus radiation group.

KC diet itself had very little effect on the animal's body weight, indicating that the diet itself was tolerable. Body weight remained very close to the starting weight in animals that were changed to KC three days following implantation. Eighteen days following implantation, body weights for SD and KC fed animals start to decline slowly as symptoms began to present (Figure 4A). Weight loss just prior to death is a function of the onset of symptoms due to tumor burden and not KC treatment.

Animals fed KC and treated with radiation saw a noticeable dip in weights 3–6 days following treatment (Figure 4B), indicating that combination therapy had an effect on body weight. These animals rapidly gained their weight back and there was no difference between the 2 groups by day 15. This treatment group also had a slightly higher level of blood BHB and a slightly lower level of blood glucose on day 6 compared to day 13. While we cannot rule out the possibility that the transient drop in weight and slightly lower glucose on day 6 contributed to the survival benefit seen when radiation and KC were combined, it is unlikely that this played a major role since tumor shrinkage continued well after the animals began to regain the lost weight. Furthermore, the remaining tumor cells did not begin to regrow after day 6 when the animals began to rapidly regain their lost weight. Mukherjee et al [14] described an increase in food consumption without a concomitant increase in weight in their mouse model beginning immediately following surgical implant of brain tumor cells. Although we did not measure food intake in individual animals, we did not see a noticeable change in food consumption in the KC-fed animals that received radiation. The transient drop in weight may be a result of the cumulative effects of therapy and dietary change. One animal in the SD+radiation group survived longer than the others in that cohort and had a persistent drop in weight. Animal behavior serves as another way to assess the animal's health and well being. All animals were observed daily and we saw no change in grooming and physical activity post treatment. This animal remained active and apparently healthy until day 150 post-implantation when its weight dropped, it showed tumor-related symptoms and was euthanized.

## Discussion

There has recently been renewed interest in the role of altered cellular metabolism in cancer, and it has been suggested that cellular metabolism may be an efficacious therapeutic target. To this end we have examined the utility of increased blood ketones on the efficacy of radiation for the treatment of glioma. KetoCal® (KC) is a commercially available ketogenic diet used in the treatment of pediatric epilepsy. Our results demonstrate that KC, when administered *ad libitum*, enhances survival and slows tumor growth in our mouse model of brain tumors. KC potentiates the effect of radiation by extending survival beyond that seen with radiation alone. Irradiated animals maintained on KC demonstrated a complete loss of tumor-based bioluminescence, suggesting tumor regression and the absence of viable tumor cells. Tumors in this cohort of animals did not recur when animals were put back on standard rodent chow.

The effectiveness of the ketogenic diet as an alternative treatment for malignant glioma was first reported by Seyfried et al [20], [21] based on the idea that while normal brain can effectively use ketones as an energy source, tumor cells cannot. Using the syngeneic CT-2A and the xenograft U87 brain tumor models, Zhou et al [19] showed that caloric restriction sufficient to cause a drop in blood glucose also significantly increased survival. Furthermore, when the ketogenic diet was given in restricted amounts this effect was more pronounced. In contrast, when the ketogenic diet or standard rodent chow was given *ad libitum* they did not find a drop in blood glucose nor did they see a significant change in survival. Recently, Maurer et al [22] used long-term human glioma cell lines and rat hippocampal neurons to analyze their utilization of ketone bodies *in vitro*. They showed that although the enzymes required to metabolize ketones are present in these glioma cells, the addition of 3-hydroxybutyrate to the culture media did not protect the cells from glucose deprivation-induced cell death, nor did it alter the cells' proliferation, migration or invasive properties. They also found that a ketogenic diet did not alter tumor growth or extend the life of mice given an orthotopic injection of LNT-229 glioma cells when compared to mice maintained on standard diet. This is in contrast to our previous work using a rodent ketogenic diet [13] and the work described in this manuscript in which a human ketogenic formulation was used (KetoCal®). The reason for this is unclear, but may have to do with differences in the diet formulations. Maurer et al [22] used a diet with a ratio of fats to carbohydrates and protein of 2.7∶1. The rodent diet we used [13] had a 6∶1 ratio and KC has a 4∶1 ratio. Furthermore, there are a number of papers in the literature demonstrating that ketones have proapoptotic [23] and chemoattractant activity [24], in contrast to the results reported by Mauer et al [22]. Thus, the response to ketones may be related, in part, to the cell line and/or model system used.

Our investigation demonstrates a significant reduction of blood glucose levels between SD and KC fed *ad libitum*. On day 6 and day 13 blood glucose levels were lower in the KC group compared to the SD group. Blood glucose levels were also lower between Rad and KC+Rad on day 6 but not on day 13. Our results did not demonstrate a correlation between circulating glucose levels and survival, suggesting that the anti-tumor effects seen are likely to be due to more than just reduced glucose levels. In addition, we did not find a change in body weight between animals fed KC *ad libitum* and animals fed SD *ad libitum* (Figure 3A). A drop in weight was seen in animals treated with KC in combination with radiation around day 6; however, animals regained this weight by day 15 (Figure 3B). No direct relationship was seen between weight loss and ketone or blood glucose levels or between blood glucose levels and survival. The KC and KC plus radiation cohort showed the longest survival without a statistical difference in either blood glucose or weight loss. This agrees with the results of our earlier work [13] and serves to further the notion that survival may be independent of blood glucose levels.

In our previous work we used a syngeneic bioluminescent intracranial tumor model to show that a ∼6∶1 (fat ∶ protein+carbohydrate) rodent KD (Bioserv F3666 diet) caused a 6 day increase in median survival of animals given unrestricted amounts of the KD (p<0.0001) [13], despite the fact that there was no measureable decrease in blood glucose. Furthermore, the dynamics of tumor growth demonstrated by *in vivo* imaging of implanted GL261-luc cells demonstrated a reduction in the rate of tumor growth in animals fed KD [13], just as we now report using KC. Molecular analyses of tumor and non-tumor tissue showed a reduction in reactive oxygen species (ROS) in the tumor from animals fed the KD. A reduction in ROS was also shown in cultured GL261 cells when ketone bodies were added to complete media *in vitro*, providing additional evidence for some efficacy even in the absence of reduced glucose.

Seyfried et al [25] suggested that radiation and chemotherapy may promote a more favorable metabolic environment (i.e. increased glucose and glutamine) for glioma growth, thus reducing long term survival. While there may be local increases in blood glucose and/or glutamine in our model system, we did not see an increase in blood glucose in the animals treated with radiation. Furthermore, we did see a highly significant increase in long term survival. The profound survival increase seen in animals treated with KC and radiation may be due to the increased radiation cytotoxicity of tumor cells as a result of sensitization by KC due to the systemic effects of this diet. Similar results have been reported in the literature. The regulation of glucose in cells treated with cisplatin and carboplatin enhanced their sensitivity [26]. Cells cultured with 2-deoxyglucose (2DG) had a 1.8 to 2.6 fold increase in cellular sensitivity to cisplatin [26]. 2DG has been proposed as a way to simulate the ketogenic diet and has been shown to enhance radiation and chemotherapeutic response [27]–[30]. Preclinical studies have served as the basis for the use of 2DG and other dietary intervention in tandem with chemotherapy and radiation [31]. Intravenous administration of 2DG 5–10 min prior to focal irradiation caused 50–60% rates of tumor-free survival (“cure”) in a number of tumor model systems [31]. Furthermore, a number of studies using Akt inhibitors have demonstrated increased radiosensitivity in cells when activation of Akt is reduced [32], [33,33], thus providing another mechanism through which the KC may be affecting radiosensitivity in these tumors.

There are a few case reports in the literature suggesting that a ketogenic diet may be an effective therapy for the treatment of human brain tumors [18], [34], [35]. These patients were not enrolled in controlled trials and institutions have utilized varying formulations of the KD; variations in adherence include restricted and unrestricted approaches as well as differing durations of implementation. A medium chain triglyceride (MCT) formulation of the ketogenic diet was implemented in 2 pediatric patients diagnosed with advanced stage malignant astrocytomas [35]. The MCT-based formulation of the ketogenic diet utilized by Nebeling et al is composed of 60% MCT oil, 20% protein, 10% carbohydrates, and 10% other dietary fats [34]. The patients tolerated the diet well and experienced notable clinical improvements 4 to 5 years after diagnosis [35]. Both patients underwent radiotherapy prior to the administration of the ketogenic diet and one of the patients also received chemotherapy. Both patients showed a decline in tumor glucose metabolism which resulted in an improved prognosis with greater median survival. More recently, Zuccoli et al [18] reported on the use of a calorically restricted ketogenic diet in a 65 year old woman diagnosed with a multifocal GBM. Two weeks after the beginning of the KD the patient received standard radiation and temozolomide treatment. Tumor regression was seen 2.5 months following diagnosis. Approximately 7 months after beginning the restricted ketogenic diet the patient stopped following the calorically restricted diet and 3 months later the tumor recurred and the patient succumbed approximately 20 months following initial diagnosis. This report demonstrated the tolerability of a reduced calorie ketogenic diet in an adult diagnosed with a GBM. In addition, the diet may have inhibited tumor growth as it is unusual for a multifocal GBM to respond to standard therapy alone in 2.5 months. Finally, it is likely that the diet suppressed edema since the patient did not receive steroids during the radiation and chemotherapy treatment and did not appear to require them. Our previous work demonstrating a reduction in the expression of the pro-inflammatory gene cyclooxygenase 2 (COX-2) supports this as well [13].

In conclusion, we demonstrated that the effect of a ketogenic diet was more than additive when used in combination with radiation for the treatment of glioma in a mouse model system. The ketogenic diet can be challenging to implement, we therefore used the commercially available ketogenic diet KetoCal® (KC) since this product is already in use for the clinical treatment of refractory epilepsy. Mice fed KC alone had increased survival compared to those fed SD. Furthermore, the combination of KC and radiation led to the absence of detectable tumor in 9 of 11 mice. This response continued even after the mice were switched back to SD 104 days following tumor implantation. With few exceptions, when carried out appropriately the diet is well tolerated in both mice and humans as demonstrated by a host of animal studies as well as human case studies. The clinical implementation of the ketogenic diet as a viable treatment modality should be seriously considered in light of our new insights into the cellular and molecular mechanisms of the diet as well as the positive response seen in the available clinical implementations.

## Materials and Methods

### Ethics Statement

This study was performed in strict accordance with the recommendations in the Guide for the Care and Use of Laboratory Animals of the National Institutes of Health. The protocol was approved by the Institutional Animal Care and Use Committee of St. Joseph's Hospital and Medical Center (protocol number 334 (A3510-01)). All surgery was performed under ketamine/xylazine anesthesia, and every effort was made to minimize suffering.

### GL261 mouse model of glioma

GL261 cells were obtained from DCTD Tumor Repository (NCI, Frederick, MD) and grown in DMEM supplemented with 10% fetal calf serum (FCS) at 37°C with 5% CO_2_. To facilitate a quantitative measurement of tumor growth rate GL261 cells were stably transfected with the gene encoding luc2 using the pGL4.51[*luc2*/CMV/Neo] vector (Promega Corp, Madison, WI) and FuGENE® 6 Transfection Reagent (Roche Applied Science, Indianapolis, IN) following conditions specified by the manufacturer. Stable transfectants were selected and maintained in Dulbecco's modified eagles medium (DMEM) containing 10% FCS and 100 µg/ml Geneticin® (G418, Invitrogen Corp, Carlsbad, CA). These cells were designated GL261-luc2 and their growth rate is equivalent to the parental GL261 cell line. Cells were harvested by trypsinization, washed and resuspended at a concentration of 1–2×10^7^ cells/ml in DMEM without FCS and implanted into ten week old C57BL/6-cBrd/cBrd/Cr (albino C57BL/6) mice (National Cancer Institute at Frederick Animal Production Program, Frederick, MD) at an average weight of 20 grams as described [13]. The implantation method has been described elsewhere [36]. Briefly, animals were anesthetized by an intraperitoneal injection of ketamine (10 mg/kg) and xylazine (80 mg/kg), placed in a stereotactic apparatus and an incision was made over the cranial midline. A burrhole was made 0.1 mm posterior to the bregma and 2.3 mm to the right of the midline. A needle was inserted to a depth of 3 mm and withdrawn 0.4 mm to a depth of 2.6 mm. Two µl of GL261-luc2 cells (10^7^ cells/ml) were infused over the course of 3 minutes. The burrhole was closed with bonewax and the incision was sutured. Mice were housed in groups of 5 in the animal care facility of St. Joseph's Hospital and Medical Center in rooms with controlled temperature and humidity under a 12-hour light-dark cycle according to the guidelines outlined in the NIH Guide for Care and Use of Laboratory Animals.

### Treatment and animal monitoring

Following surgery, animals were fed standard rodent chow for 3 days. Animals were then randomized to remain on standard rodent chow (SD) *ad libitum* or changed to KetoCal® (KC) *ad libitum*. KetoCal® was obtained directly from the manufacturer and is a nutritionally complete diet providing a 4∶1 ratio of fats to carbohydrates plus protein (72% fat, 15% protein, and 3% carbohydrate; for complete nutritional information see http://www.shsna.com/pages/ketocal41.htm). A paste was prepared by mixing KetoCal® powder with water (2∶1). Animals in each cage received a cubic inch of the paste each day which was sufficient to provide *ad libitum* feeding.

On days 3 and 5 post-implantation animals receiving radiation were given an intraperitoneal injection of xylazine (10 mg/kg) and ketamine (80 mg/kg), shielded and positioned in the RS 2000 X-Ray Biological Irradiator (Rad Source Technologies, Suwanee, GA) such that only their head received 4 Gy of radiation at 2 Gy per minute. Animals received two fractions of radiation (4 Gy each) to more closely approximate the fractionated course of treatment that patients would undergo [37].

Bioluminescence was analyzed every third day to quantify tumor burden [13]. Animals received a subcutaneous (s.c.) injection of 150 µg luciferin/kg body weight 15 min prior to *in vivo* imaging using an IVIS® Spectrum *in vivo* imaging system (Caliper Life Sciences, Hopkinton, MA). Tumor cells were detectable 3 days post implantation (the first day they were imaged) and quantitation was done using the system's Living Image® 3.1 software.

Serum β-hydroxybutyrate levels were measured using a Keto-Site reflectance meter (GDS Diagnostics, Elkhart, IN) and blood glucose levels were tested using a HemoCue Glucose 201 System (HemoCue USA, Lake Forest, CA) on blood obtained from tail clips as described [13]. Animals were weighed every 3 days to ensure that all the animals were gaining weight in an equivalent manner. Animals were euthanized upon occurrence of visible symptoms of impending death such as hunched posture, reduced mobility and weight loss [13], [38].

### Statistical Analysis

Survival data was analyzed using Kaplan-Meier plots and log-rank statistics (GraphPad Prism® v 5.04, GraphPad Software, San Diego, CA). Survival data from SD alone, KC alone, SD+radiation, and KC+radiation were examined for interaction effects using Cox Proportional Hazards. The model examined SD and KC in the context of radiation and radiation+KC. The method used right censoring, robust variance estimates, and Breslow's method for ties with a convergence tolerance of 0.0001, allowing colinearity.
